# Gallstone Frequency in Children With Celiac Disease

**DOI:** 10.7759/cureus.12767

**Published:** 2021-01-18

**Authors:** Mehmet Agin, Yusuf Kayar

**Affiliations:** 1 Pediatric Gastroenterology, Van Education and Research Hospital, Van, TUR; 2 Gastroenterology, Van Education and Research Hospital, Van, TUR

**Keywords:** celiac disease, gallstone, ultrasonography

## Abstract

Introduction

Celiac disease (CD) is a chronic autoimmune systemic disease caused by the T cell-driven immune mechanism, which is triggered by gluten in cereals such as wheat, barley, and rye in individuals who have a genetic predisposition. The purpose of the present study was to investigate the frequency of gallstones in children with CD.

Methods

A total of 120 patients who were diagnosed with CD and who were followed-up by the pediatric gastroenterology clinic of the hospital and 100 healthy children were included in the study. The age, gender, hemogram, biochemistry, and abdominal ultrasonography images of the patients were compared. Cases that had gallstones were evaluated in terms of fasting serum lipids, glucose-6-P dehydrogenase, and pyruvate kinase, osmotic fragility, hemoglobin electrophoresis, and peripheral smears. Cases diagnosed with a hematological or metabolic disease were excluded from the study. Celiac serology was examined in terms of CD diagnosis in cases who had gallstones for the first time.

Results

The median age of the patients with CD who were included in the study was eight years (5-12), and the median age of the control group was 10 years (6-13). A total of 48% of the Control Group was female, and 52% were male. No significant differences were detected between the age and gender distribution of the cases. There were no differences between hemogram and biochemical parameters. Gallstones were detected in six (5%) of CD-diagnosed cases, and in three (3%) of the cases in the control group. Two (2/160; 1.3%) of the patients who were referred to our clinic with the diagnosis of gallstones were diagnosed with CD.

Conclusions: Early diagnosis and treatment of CD is important to avoid gallstone development because a gluten-free diet corrects enteropathy significantly in CD. CD must be considered in cases with gallstones.

## Introduction

Celiac disease (CD) is a chronic autoimmune systemic disease caused by T cell-mediated immune mechanism, which is triggered by gluten in cereals, such as wheat, barley, and rye in individuals who have a genetic predisposition. Although the frequency of the disease varies among geographical areas, the highest incidence is detected in countries, such as Turkey, Western Europe, North America, and Australia, where wheat has an important place in nutrition. Environmental, immunological, and genetic factors play roles in its pathogenesis [1-3].

Although many autoimmune diseases may accompany celiac disease, it can present with many extra-intestinal findings other than intestinal symptoms [4-5]. Especially, hepatobiliary and pancreatic involvement are common [6]. Autoimmune diseases originating from the liver may occur as a result of common genetic factors and common immunopathogenesis, as well as problems in nutrient absorption secondary to malnutrition, fat in the liver, and negatively affecting the release of pancreatic exocrine hormones [6]. However, although the relations between celiac disease, hepatobiliary tract, and pancreas is clear, the relation between celiac disease and gallstone formation is still not clear.

It was reported approximately 45 years ago that cholecystokinin (CCK) secretion from the proximal small intestine decreased because of the gluten enteropathy in patients with CD, and the emptying of the gallbladder was impaired dramatically [7]. CCK is a hormone that is synthesized and secreted by I-cells from the proximal small intestine mucosal epithelium in response to a meal with fat and protein [8]. After CCK is secreted, it enters the circulation and stimulates the contraction of the gallbladder and the relaxation of the Oddi sphincter. This then stimulates the release of bile in the gallbladder into the small intestine [9]. Before a gluten-free diet (GFD), the gallbladder becomes large, lax, and lazy in celiac disease patients [10], which poses a risk for the formation of biliary sludge and gallstones. Unfortunately, whether these patients are prone to the formation of gallstones still remains a matter of debate. In the literature, although there are very few studies on the adult age group on this subject, there are no epidemiological or clinical trials regarding childhood CD. In the present study, the purpose was to investigate the frequency of gallstones in children with CD.

## Materials and methods

Selection of patients

A total of 120 patients between the ages of two and 18, diagnosed with celiac disease based on the European Society for Paediatric Gastroenterology Hepatology and Nutrition (ESPGHAN) criteria followed-up by the children’s gastroenterology clinic at the University of Health Sciences, Van Education and Research Hospital, and 100 patients who did not have CD were included in the study. The files of the patients were examined retrospectively. The age, gender, hemogram, biochemistry, and abdominal ultrasonography imaging (USG) results of the patients were examined. The cases that had gallstones were evaluated in terms of fasting serum lipids, glucose-6-P dehydrogenase and pyruvate kinase, osmotic fragility, hemoglobin electrophoresis, and peripheral smears. The cases diagnosed with a hematological or metabolic disease were excluded from the study.

The celiac serology tests of 160 patients with gallstones referred to our clinic were also examined. Upper gastrointestinal tract endoscopy was performed on the patients who had high celiac serology results, and biopsies were taken from the duodenum for CD diagnosis.

Endoscopic evaluation

Endoscopy of the patients was performed by using the Fujinon EG530WR endoscopy device (Fujifilm Europe, Duesseldorf, Germany) in the endoscopy unit of Van Training and Research Hospital. Verbal and written consent was received from the families before the endoscopy. Hunger was achieved for six hours in all patients before the endoscopy, and the endoscopic procedure was performed after local pharyngeal xylocaine anesthesia when the patients were sedated with midazolam at 0.1 mg/kg and ketamine at 1 mg/kg. The duodenum was examined thoroughly during the endoscopy, and multiple biopsies were taken from different locations as reported previously for the diagnosis of CD.

Histopathologic evaluation

Histopathologic evaluation was made according to the Marsh classification. The duodenal biopsies taken endoscopically were sent to the pathology laboratory in 10% formaldehyde. After routine tissue follow-up processes, the tissue samples that were embedded in paraffin were cut at 5-micron thickness, stained with routine Hematoxylin-Eosin (H-E) staining, and were then evaluated by a light microscope. CD diagnosis was made based on characteristic histological findings (i.e., increased intraepithelial lymphocytes, villous atrophy, and crypt hyperplasia) that were classified according to the standard classification as suggested by Marsh [2,11].

Ultrasonographic evaluation

USG was performed after fasting during the night. All USG procedures were performed by three independent radiologists. The patients for whom the three physicians provided the same reports were included in the study. The definition of cholelithiasis was made based on detecting echogenic structures that had acoustic shadows in the visible gallbladder. The lumen or hepatocholedochus duct, one or more echogenic structures in the gallbladder (without dorsal shadow), or a structure that had echogenicity and a dorsal acoustic shadow in the gallbladder did not show adequate visualization in the lumen.

Ethical declaration

All participants provided written permissions to participate in the study. Ethical approval was received from the Ethics Committee of our hospital (Van, Turkey) to conduct this study. All procedures were in line with the ethical standards of the human trials committee of our institution and the Helsinki Declaration. Written informed consent forms were obtained from all participants who were evaluated by a gastroenterologist and then included in the study.

Statistical method

The normality of distribution of the continuous variables was tested by the Shapiro-Wilk test. The Mann-Whitney U-test was used to compare non-normal numerical data between the two groups. Fisher’s exact test was applied to test the relationships between categorical variables. Statistical analysis was performed with the Statistical Package for the Social Sciences (SPSS) for Windows version 24.0 (IBM Corp., Armonk, NY); and a p-value of <0.05 was accepted as statistically significant.

## Results

The median age of the patients who had CD was eight years (minimum 5 years - maximum 12 years), and the median age of the control group was 10 years (minimum 6 years - maximum 13 years). A total of 57% (68/120) of the cases with CD was female and 43% (52/120) were male. A total of 48% (48/100) of the control group were female, and 52% (52/100) were male. No significant differences were detected between the age and gender distribution of the cases. No differences were found between the hemogram and biochemical parameters. Two of the 160 patients who were detected to have gallstones and were referred to us from other healthcare centers were diagnosed with CD endoscopically and histopathologically after celiac serology was positive. Gallstones were detected in six (5%) of the cases diagnosed with CD and in three (3%) of the cases in the control group. The four cases who had gallstones (4/120; 3.3%) were followed up with CD, the other two (2/160; 1.3%) cases were those with gallstones and were referred to our clinic with the diagnosis of CD in histopathological examination (Table 1).

**Table 1:** Comparison of demographic, laboratory, and imaging results of the celiac disease and control group

	Celiac Disease (N = 120)	Control (N = 100)	P-value
Variables	n (%)	n (%)	
Gender			0.174
Female	68 (57%)	48 (48%)	
Male	52 (43%)	52 (52%)	
Presence of Gallstone	6 (5%)	3 (3%)	0.456
	Median (min-max)	Median (min-max)	
Diagnosis age (Years)	8 (5 -12 )	10 (6 -13 )	0.227
Leucocyte count (Range: 4.5-1.5x10^3^/µL)	8280 (6880 -10100 )	8000 (6620 -9600 )	0.235
Hemoglobin (Range: 12-16 g/dl)	12.1 (11.1 -13.6 )	12 (11 -13.1 )	0.950
Platelets (Range:150-450x10^3^/µL)	342000 (283000 -388000 )	331000 (299000 -379000 )	0.899

The serum lipids, glucose-6-P dehydrogenase and pyruvate kinase, and osmotic fragility of the cases who had gall bladder stones in abdominal USG were evaluated with hemoglobin electrophoresis, hemogram, and peripheral smear, and no pathologies were detected.

## Discussion

The prevalence and etiology of gallstones in the child age group were reported at different rates in different countries and regions [12]. Gallstone prevalence was reported to be 0.15% in 1959 in children and was 1.9% in a study conducted in 2000 [13-14]. Although the incidence of childhood gallstones is less than that of the general population, its prevalence is increasing. With the development and increasing spread of imaging methods, such as USG, the diagnosis of gallstones began to increase gradually when compared to previous years [15].

It was reported that the plasma levels of hormones such as neurotensin, CCK, and somatostatin changed in patients with CD due to proximal intestinal enteropathy, and depending on this, disorders appear in gallbladder fasting volume and gallbladder contraction and discharge [7,16-18]. As known for a long time, plasma amino acid levels may show differences in regard to CD. Given that amino acids are potent inhibitors of bile acid uptake, alteration of plasma amino acid levels may cause stone formation due to impaired flow in the biliary tract [19]. Whether these patients are inclined to have gallstone formation is a matter of debate. It was reported in previous studies that hormone neurotensin increased in untreated celiac disease patients. It was also reported that the increase of this hormone might delay the emptying of the stomach, and disrupt the motility of the gallbladder directly or indirectly [18]. It was reported in the study conducted by Fraquelli et al. that the gallbladder volume and plasma somatostatin levels were more at the time of diagnosis in CD patients than in the control group, and these findings improved completely after GFD [20].

In patients with CD, postprandial CCK levels are low despite the increased number of cells secreting duodenal CCK. As a result of impaired exogenous fat lipolysis, cells secreting CCK are not sufficiently stimulated for CCK release. The inactivity of the gallbladder contributes to the enterohepatic circulation of bile salts and the deterioration of small bowel transit time [21]. Interestingly, gastrointestinal hormone anomalies and their effects on the gallbladder, stomach, and small intestine motility become completely normal following GFD [20,22-24]. It was reported in another study that plasma CCK levels and I-cell counts following GFD became normal [25].

These classic observations show that patients with CD are prone to the formation of gallstones before GFD begins. In the present study, we detected gallstones in six patients with CD diagnosis (5%) and in three (3%) patients in the control group. In our study, although no statistically significant differences were detected between CD-diagnosed cases and the control group, the frequency of gallstones increased in patients with CD compared to the normal population. The most accurate information we know about this was reported in a study conducted in the adult age group. In this study, the prevalence of gallstones was reported to be 20% in patients with CD diagnosed with intestinal biopsy and was 12% in non-CD cases [26]. Since GFD begins immediately after the diagnosis of patients with CD, it is difficult to say anything about the prevalence of gallstones in patients with CD.

In a study investigating the etiology of cholelithiasis and choledocholithiasis, CD was detected in only one in 98 child patients as a comorbidity [27]. Two (1.3%) of the 160 children who were referred to our clinic with a gallstone diagnosis were diagnosed as CD endoscopically and serologically during our study period.

It is not clear whether patients who are diagnosed with gallstones with USG or who underwent cholecystectomy due to gallstones were CD or not. This causes that the prevalence of real gallstones is overlooked in cases with CD. Also, there is no clear data showing the actual prevalence of CD because no CD is considered in the foreground when CD is asymptomatic, atypical, or sometimes mild in the children age group [28]. Some celiac disease patients do not respond well to GFD; and for this reason, they must be followed up in terms of the presence of gallstones with USG both at the time of diagnosis and in follow-ups regardless of whether they receive GFD or not.

Our study has strengths and weaknesses. The retrospective design of our study may reflect the rate of gallstones accompanying CD as lower due to the likelihood of gallstones that may develop in the follow-ups of these patients. However, the sufficient number of patients included in the study, and the contribution of it to the hypothesis that the frequency of gallstones may be more in CD than in the normal population is also one of the strengths of our study.

## Conclusions

As a result, since GFD corrects enteropathy in CD at significant levels, the early diagnosis and treatment of CD is important in preventing the development of gallstones. Patients with CD must be followed up in terms of gallstones with USG both at the time of diagnosis and in follow-ups, regardless of whether they receive GFD or not. Also, CD must be questioned in cases when gallstones are detected. However, multicentric studies with wider participation designed prospectively are needed to clearly reveal the relation between gallstone formation and CD.
